# Metformin induces an intracellular reductive state that protects oesophageal squamous cell carcinoma cells against cisplatin but not copper-bis(thiosemicarbazones)

**DOI:** 10.1186/1471-2407-14-314

**Published:** 2014-05-05

**Authors:** Leonard Howard Damelin, Rupal Jivan, Robin Bruce Veale, Amanda Louise Rousseau, Demetra Mavri-Damelin

**Affiliations:** 1 School of Pathology, Faculty of Health Sciences, University of the Witwatersrand, 7 York Road, Parktown, Johannesburg 2193, South Africa; 2 Cell Biology Group, Centre for HIV and STI’s, National Institute for Communicable Diseases, Private Bag X4, Sandringham, Johannesburg 2131, South Africa; 3 School of Molecular and Cell Biology, University of the Witwatersrand, Private Bag X3, Johannesburg 2050, South Africa; 4 Molecular Sciences Institute, School of Chemistry, University of the Witwatersrand, Private Bag X3, Johannesburg 2050, South Africa

**Keywords:** Oesophageal squamous cell carcinoma, Metformin, Copper bis(thiosemicarbazones), Metabolism, Cisplatin, Thiol, Glutathione

## Abstract

**Background:**

Oesophageal squamous cell carcinoma (OSCC) is a highly aggressive carcinoma with a poor survival rate. One of the most commonly used chemotherapeutic drugs, cisplatin, displays varied and often poor efficacy in vivo. Therefore, alternative, cost-effective and more efficacious treatments are required. Metformin has been previously shown to reduce proliferative rates in various carcinoma cell lines. We report for the first time, the effect of metformin on OSCC cell proliferation and show that it antagonises cisplatin-induced but not copper-bis(thiosemicarbazone)-induced cytotoxicity in OSCC cells.

**Methods:**

Cell proliferation and stage of the cell cycle were quantified by trypan blue counts and flow cytometry, respectively. All cytotoxicity measurements were made using the tetrazolium based MTT assay. Metabolic alterations to cells were determined as follows: glycolysis via a lactate dehydrogenase assay, reducing equivalents by MTT reduction and reduced intracellular thiols by monobromobimane-thiol fluorescence, and glutathione depletion using buthionine sulfoximine. Inductively coupled plasma mass spectrometry was used to quantify cisplatin-DNA adduct formation.

**Results:**

Metformin was found to reduce cell proliferation significantly in all OSCC cell lines, with an accumulation of cells in G0/G1 phase of the cell cycle. However, metformin significantly protected OSCC cells against cisplatin toxicity. Our results indicate that a major mechanism of metformin-induced cisplatin resistance results from a significant increase in glycolysis, intracellular NAD(P)H levels with a concomitant increase in reduced intracellular thiols, leading to decreased cisplatin-DNA adduct formation. The glutathione synthesis inhibitor buthionine sulfoximine significantly ablated the protective effect of metformin. We subsequently show that the copper-bis(thiosemicarbazones), Cu-ATSM and Cu-GTSM, which are trapped in cells under reducing conditions, cause significant OSCC cytotoxicity, both alone and in combination with metformin.

**Conclusions:**

This is the first study showing that metformin can be used to decrease cell proliferation in OSCC cells. However, metformin protects against cisplatin cytotoxicity by inducing a reducing intracellular environment leading to lower cisplatin-DNA adduct formation. As such, we advise that caution be used when administering cisplatin to diabetic patients treated with metformin. Furthermore, we propose a novel combination therapy approach for OSCC that utilises metformin with metformin-compatible cytotoxic agents, such as the copper-bis(thiosemicarbazones), Cu-ATSM and Cu-GTSM.

## Background

Oesophageal carcinoma, of which there are two subtypes, adenocarcinoma and squamous cell carcinoma, is the sixth most common cause of cancer-related death [1]. Oesophageal squamous cell carcinoma (OSCC) is a highly aggressive carcinoma with a very poor survival rate that occurs with particularly high frequency in developing countries including Iran, China, South Africa, and Brazil, where mortality rates can exceed 100 per 100,000 population. In developed countries, incidence rarely exceeds 10 per 100,000 population [2,3], with the exceptions of certain regions in North-West France and Northern Italy where incidence may reach 30 and 2 per 100,000 in males and females, respectively [4]. The causes of OSCC are multiple and varied, probably reflecting repeated exposure to dietary components, such as N-nitroso compounds, excessive smoking and alcohol consumption, chronic inflammation and possibly, genetic predisposition [5]. Current commonly used therapies for OSCC include 5-fluorouracil and cisplatin, which show poor efficacy and often display both chemotoxicity and chemoresistance [6]. Cisplatin has multiple mechanisms of cytotoxicity including the formation of DNA and protein adducts, as well as via oxidative stress. Many resistance mechanisms for cisplatin have been identified, including, pertinent to this study, sequestering of cisplatin by glutathione (a major species of intracellular thiols). This results in export of cisplatin-glutathione adducts leading to a reduction in cisplatin-mediated DNA damage [7-9]. Investigations into more effective targeted treatment options for OSCC using monoclonal antibody therapies are very promising [10] however, access to such therapies in developing countries is extremely limited, primarily due to cost. Therefore, there is a continued and urgent requirement for alternative, effective and economical treatment options.

Recently, the well characterized and tolerated anti-diabetic drug, metformin has been the subject of intense investigations in cancer research. Population studies have shown that this biguanide, conventionally used to decrease peripheral glucose levels and increase insulin sensitivity in diabetic and pre-diabetic patients [11,12], reduced breast cancer occurrence in female patients with type 2 diabetes [13]. Since then, metformin has been observed to reduce the proliferation of many types of carcinoma cell lines and diabetic patients taking metformin have been found to have better recovery rates from breast cancer [14-17]. Furthermore, metformin has been shown to target cancer stem cells [18]. However, whilst metformin reduces cell proliferation in most cancer types, it rarely causes apoptosis, and is therefore being combined with conventional chemotherapeutic drugs, including cisplatin. This treatment combination has mixed results, with some studies showing that metformin can enhance the effectiveness of chemotherapeutic drugs whilst others have shown increased chemoresistance in the presence of metformin [19,20]. With regards to cisplatin, metformin has been shown to reduce cisplatin sensitivity through the AMPK-independent upregulation of the Akt survival pathway [20]. A search on *clinicaltrials.gov* found over 40 clinical trials investigating metformin and a variety of chemotherapeutic drugs, for breast, ovarian and prostate cancer amongst a number of others.

In this study, we investigated the effect of metformin on OSCC cell proliferation and on the cytotoxicity of cisplatin for OSCC cells. We show that whilst metformin markedly reduces OSCC cell proliferation and causes cells to accumulate in the G0/G1 phase of the cell cycle, it also significantly protects against cisplatin cytotoxicity. The protective effect is not solely due to reduced cell-proliferation, as the biguanide minimally to partially protects against the DNA-crosslinker, mitomycin C, but is dependent on a metformin-induced increase in glycolysis and intracellular NAD(P)H levels with a concomitant increase in reduced intracellular thiols, which coincides with decreased cisplatin-DNA adduct formation. The glutathione synthesis inhibitor buthionine sulfoximine (BSO) significantly reverses this protective effect, confirming the role of reduced glutathione in cisplatin detoxification by metformin-treated cells. In light of these findings, we investigated the copper-bis(thiosemicarbazones), copper diacetyl-bis(4-methylthiosemicarbazonato)copper(II) (Cu-ATSM) and copper glyoxal-bis(4-methylthiosemicarbazonato)copper(II) (Cu-GTSM). Copper-bis(thiosemicarbazones) induce cytotoxicity through a number of mechanisms, including inhibition of DNA synthesis [21]. Importantly, as these compounds are known to be trapped in cells under reducing conditions, they are therefore compatible with a reducing intracellular state [22]. We show that both Cu-ATSM and Cu-GTSM display significant levels of cytotoxicity at LD_50_ values comparable to or lower than cisplatin, both alone or in combination with metformin, highlighting the use of metformin and reduction-compatible cytotoxic drugs as a novel combination therapy strategy for the treatment of OSCC.

## Methods

### Reagents

Reagents for flow cytometry were purchased from Beckman Coulter. All other reagents were purchased from Sigma Aldrich unless otherwise specified.

### Synthesis of bis(thiosemicarbazones)

The bis(thiosemicarbazones), ATSM and GTSM, were synthesised from 4-methyl thiosemicarbazide and butanedione or glyoxal, respectively, according to the method of French *et al. *[23].

ATSM: ^1^H NMR (500 MHz, DMSO) 11.74 (2H, s, 2 × C*H* = N), 8.48 (2H, d, *J* = 4.2, 2 × NH), 7.72 (2H, s, 2 × NH), 2.96 (6H, d, *J* = 4.4, 2 × CH_3_); ^13^C NMR (126 MHz, DMSO) 177.55 (2 × C = S), 140.02 (2 × C = N), 30.89 (2 × CH_3_).

GTSM: ^1^H NMR (500 MHz, DMSO) 10.20 (2H, s, 2 × NH), 8.36 (2H, d, *J* = 4.1, 2 × NH), 3.02 (6H, d, *J* = 4.5, 2 × CH_3_), 2.20 (6H, s, 2 × CH_3_); ^13^C NMR (126 MHz, DMSO) 178.47 (2 × C = S), 147.95 (2 × C = N), 31.18 (2 × CH_3_), 11.64 (2 × CH_3_).

Cu-ATSM and Cu-GTSM were synthesized from ATSM and GTSM and cupric chloride as previously described [24].

### Cell culture

The human OSCC cell lines were a kind gift from Professor Robin Veale. These cells, WHCO1, WHCO5 [25] and SNO [26] were maintained in Dulbecco’s Modified Eagles Medium/Hams F12 (DMEM/Hams F12, 3:1) supplemented with 10% FCS at 37°C and 5% CO_2_.

### Cell proliferation

Cell proliferation was assessed by cell counts using trypan blue exclusion. Cells were seeded in 48-well plates at 1×10^4^ cells per well. After 24 hours, cells were incubated with or without 10 mM metformin for an additional 24 hours. Cells were then trypsinized, resuspended in 1×PBS and incubated in 2% trypan blue for 2 minutes and counted using a haemocytometer (n = 5 ± SD).

### Cell cycle analysis

Cell cycle analysis was by flow cytometry as previously described [27]. Cells were seeded equally in 10 cm dishes and cultured for 48 hours (~60% confluent). At this time, the medium was replaced and cells incubated with or without 10 mM metformin for 24 hours. Control cells were serum-deprived for 8 hours. Cells were then harvested and prepared for analysis using the DNA Prep Reagent kit according to manufacturers’ instructions (Beckman Coulter). Briefly, cells were treated with DNA prep LPR (lysis) solution in order to facilitate propidium iodide (PI) entry and samples vortexed for 10 seconds followed by the addition of DNA prep stain (PI and RNAse) and additional vortexing. Samples were then immediately analysed on an LSRFortessa™ cell analyser, BD Biosciences. DNA histograms were analysed using FlowJo v10 software and the percentage of cells in the G0/G1, S, and G2/M phase of the cell cycle calculated (n = 3 ± SD).

### Cytotoxicity assays

Cytotoxicity was assessed using the 3-(4,5-dimethylthiazol-2-yl)-2,5 diphenyltetrazolium bromide (MTT) assay. Cells (8500 cells per well) were seeded into 96-well plates and after 24 hours exposed to cytotoxic agents for varying times. After treatment, the medium was replaced with 100 μl of MTT solution (0.5 mg/ml in cell culture medium) and incubated at 37°C for 2 hours. MTT solution was then removed, and MTT formazan dissolved in 100 μl dimethyl sulfoxide (DMSO). Absorbance was measured at 570 nm using the Bio-Rad iMark microplate reader (n = 3 ± SD).

### ICP-MS analysis of platinum-DNA adducts

Inductively-coupled plasma mass spectrometry (ICP-MS) was performed as previously described [28]. Briefly, cells were treated with cisplatin (LD_30_ concentrations) for 48 hours with or without 24 hour prior exposure to 10 mM metformin. Total genomic DNA was extracted, resuspended in water and quantified using a NanoDrop ND-1000 spectrophotometer (Thermo Scientific). DNA samples were hydrolysed in a final concentration of 1% HNO_3_ at 70°C for 24 hours and analysed for platinum (n = 3 ± SD) on an Agilent 7700 ICP-MS. The instrument was optimised for sensitivity and low oxides. Analysis was done in no-gas mode, and the instrument was calibrated for platinum analysis using National Institute of Standards and Technology traceable standards.

### Determination of glycolysis via lactate production

As an indicator of levels of glycolysis, lactate levels in culture medium were quantified using a lactate dehydrogenase assay [29] where the production of NADH from NAD via the conversion of lactate to pyruvate is directly proportional to lactate concentration. Cells were seeded and treated as for cell cycle analysis and both conditioned culture medium and cells collected after 24 hours. Cells were counted using trypan blue exclusion as described above. For lactate quantification, 50 μl of medium was added to 950 μl glycine-hydrazine buffer (0.64 M glycine, 0.64 M hydrazine, 4.8 mM NAD^+^, 16 U/ml lactate dehydrogenase, pH 9.2) and incubated at 37°C for 2 minutes. NADH was then quantified spectrophotometrically at 340 nm and values corrected for cell number (n = 3 ± SD).

### Quantification of reducing equivalents

Total cellular reducing equivalents were quantified by tetrazolium (MTT) assay as previously described [30]. An equal number of cells were seeded into 96-well plates (8500 cells per well) and after 24 hours cells were incubated for 24 hours with or without 10 mM metformin and MTT assay performed. Values were corrected for cell number using trypan blue exclusion as described above (n = 3 ± SD).

### Low-molecular weight thiol quantification

Total low-molecular weight thiols were quantified using monobromobimane, which forms fluorescent thiol conjugates [31]. Cells were seeded and treated as for cell cycle analysis with or without 10 mM metformin for 24 hours. Cells were subsequently washed three times with 1×PBS and incubated in 1 ml of 1×PBS with 18 μl monobromobimane solution (stock 5 mg/ml in DMSO) for 10 minutes. Cells were then lysed in 1 ml of triple detergent lysis buffer (50 mM Tris–HCl, 150 mM NaCl, 0.1% SDS, 1% Triton ×-100, 0.5% sodium deoxycholate), the lysate centrifuged at 10000 g and 100 μl of the resultant supernatant fluorescently analysed at 360nm_excitation_/460nm_emission_ using an Ascent multi-well plate fluorimeter (Thermo Scientific). Cells seeded in parallel dishes were counted using trypan blue exclusion as above and fluorescence values were corrected for cell number (n = 3 ± SD).

### Glutathione depletion assay

The glutathione synthesis inhibitor BSO was used in order to deplete intracellular glutathione levels and thereby assess the involvement of thiols (glutathione) in the cytoprotective effects of metformin on cisplatin toxicity [32]. Cytotoxicity assays were performed as above with the following modifications, cells were seeded at 7500 cells per well in 96-well plates and allowed to settle for 18 hours. Cells were then treated with 0.4 mM of BSO for 18 hours, followed by the addition of 10 mM metformin (or metformin diluent for control cells) and subsequently cisplatin, and cytotoxicity determined by MTT assay as above (n = 3 ± SD).

### Statistical analysis

Comparisons were by two-tailed Student’s t-tests and p < 0.05 was considered statistically significant. LD_50_ and LD_30_ values were calculated using GraphPad Prism version 6.

## Results

### OSCC cells exhibit decreased cell proliferation and cell cycle arrest in response to metformin

**Figure 1:**
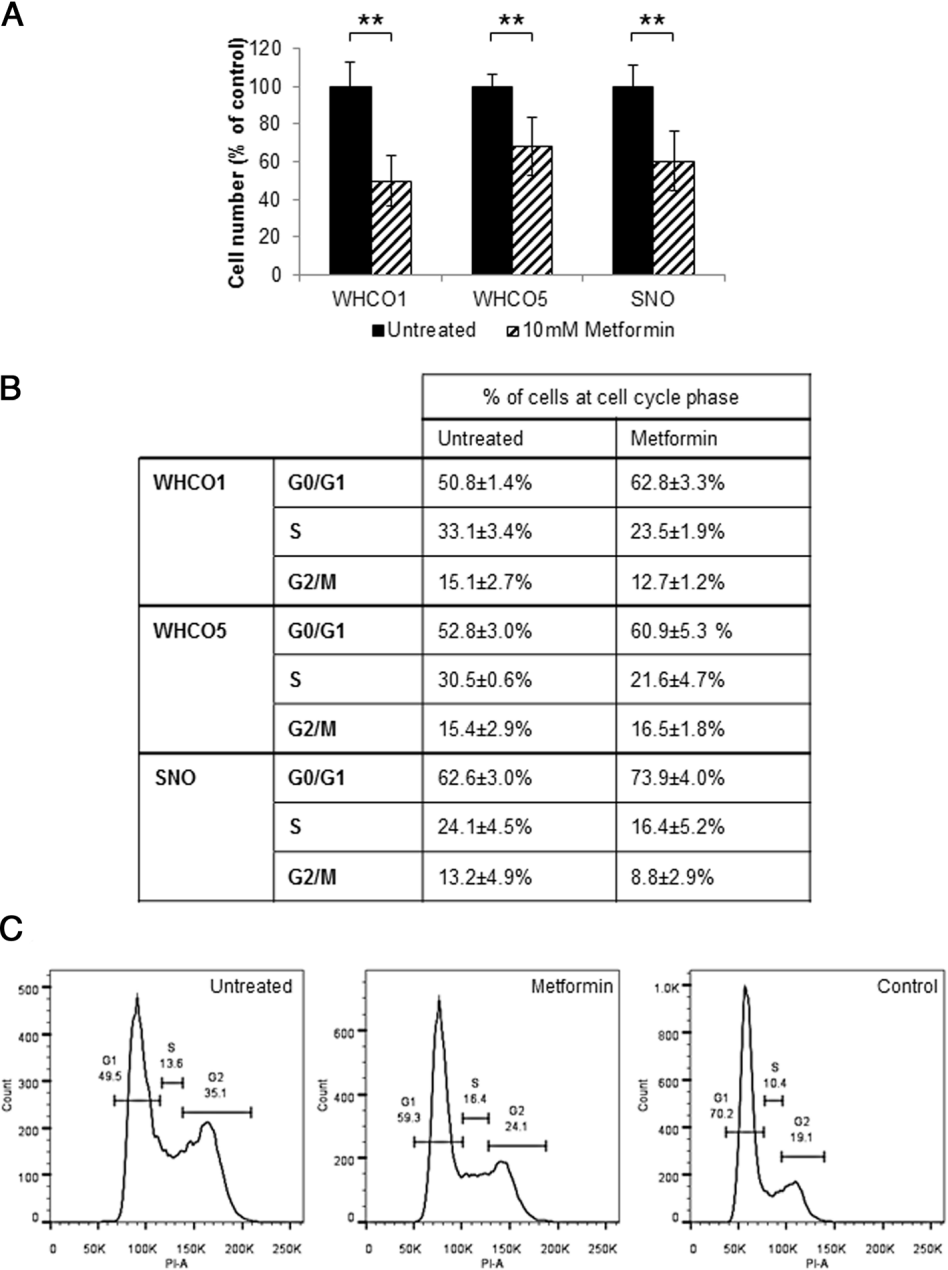
**Anti-proliferative effects of metformin on OSCC cells. A**, Cells exposed to 10 mM metformin for 24 hours showed a decrease in cell number in comparison to untreated controls across all cell lines, n = 4, mean ± SD. **B**, Quantification and **C**,representative figures of flow cytometry analysis (from SNO cells) for untreated cells (Untreated), FCS deprived control (FCS Control) and cells exposed to 10 mM metformin for 24 hours (Metformin). Metformin treated cells exhibited an accumulation at the G0/G1 stage of the cell cycle across all cell lines, expressed as % of cells in phase of cell cycle (n = 3, mean ± SD), where for all cells there was a statistically significant increase in cells in G0/G1 in metformin treated cells relative to untreated controls with WHCO1 p = 0.05, WHCO5 p = 0.04 and SNO p = 0.01.

We investigated the effect of metformin on three OSCC cell lines (WHCO1, WHCO5 and SNO), previously derived from South African OSCC patients [25,26]. All cell lines exhibited a significant reduction in cell proliferation in response to 10 mM metformin after 24 hour treatment, in comparison to untreated controls. There was 50%, 32% and 39% reduction in cell proliferation in WHCO1, WHCO5 and SNO cells, respectively (Figure 1A). In addition, we assessed cell cycle progression using flow cytometry with propidium iodide staining of cellular DNA content. Cells deprived of foetal calf serum (FCS) for 8 hours were used as the control, which as expected, showed an increase in the number of cells in G0/G1 phase of the cell cycle. Metformin treatment (10 mM for 24 hours), as anticipated, caused an increase in the number of cells in G0/G1 phase relative to untreated controls (Figure 1B and C).

### Metformin protects cells from cisplatin cytotoxicity

**Figure 2:**
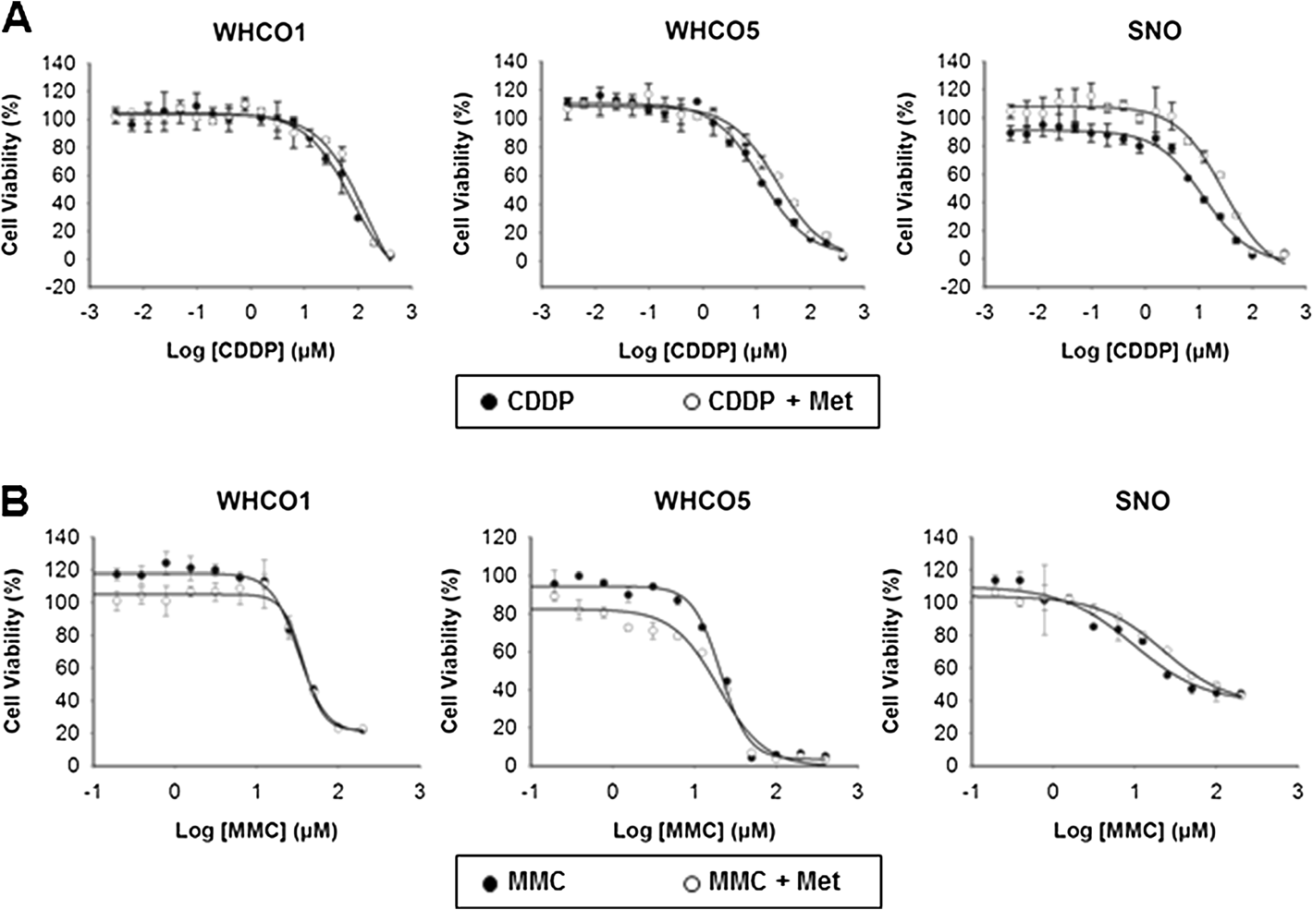
**Effect of metformin on cisplatin and mitomycin C cytotoxicity for OSCC cells.** OSCC cells, untreated or treated with 10 mM metformin for 24 hours and then treated with **(A)** cisplatin or **(B)** mitomycin C for a further 48 hours, were assessed by MTT assay. All metformin-cisplatin treated cells displayed a trend for higher LD_50_ values, with WHCO1 and SNO cells statistically higher (n = 3, mean ± SD).

**Figure 3:**
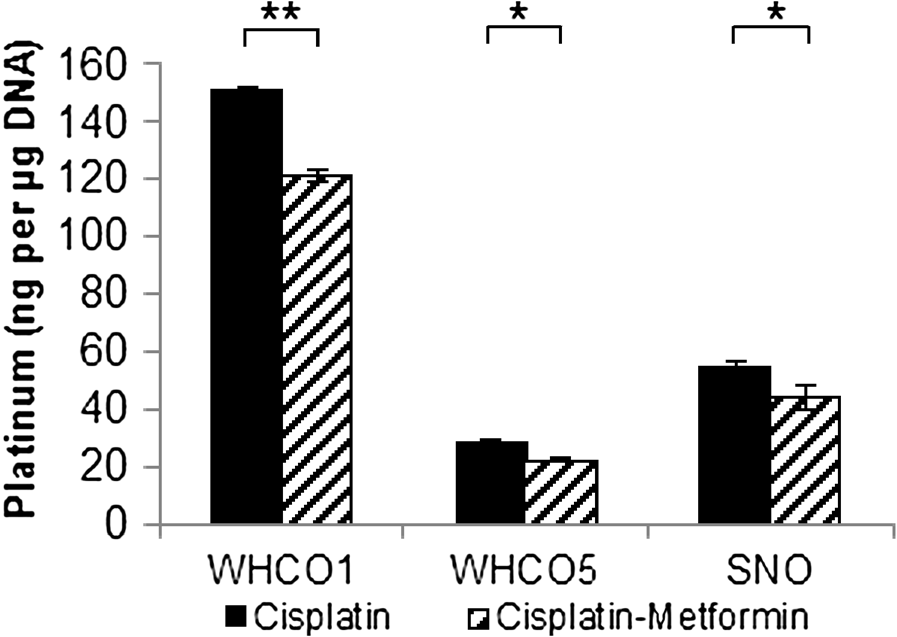
**Decreased platinum-DNA adduct formation in cisplatin-metformin treated OSCC cells.** OSCC cells were treated with LD_30_ concentrations of cisplatin, either alone or in combination with 10 mM metformin. Genomic DNA was extracted and platinum quantified by ICP-MS which showed a decrease in platinum in cisplatin-metformin treated cells in comparison to cells treated with cisplatin alone (values expressed per μg of DNA) (n = 3, mean ± SD).

Next, we assessed the effect of metformin on cisplatin cytotoxicity by MTT assay. Cells pre-treated with 10 mM metformin for 24 hours and then treated with 10 mM metformin and cisplatin for 48 hours (Figure 2A), exhibited significantly lower cytotoxicity than cells treated with cisplatin alone; thus displaying higher LD_50_ values for cisplatin in the presence of metformin, with a 78% increase for WHCO1, 140% increase for WHCO5 and 156% increase for SNO cells (Table 1). We assessed the effects of metformin on the formation of cisplatin-DNA adducts, by treating cells as above but using the calculated LD_30_ of cisplatin. DNA-bound platinum, as quantified by ICP-MS, showed a significant reduction in metformin treated cells by 19.3% in WHCO1, 14.1% in WHCO5 and 18.4% in SNO cells (Figure 3). To determine whether reduced cytotoxicity and cisplatin-DNA adduct levels was principally due to the observed metformin-induced reduction in cell proliferation, cells were treated with an alternative DNA crosslinker, mitomycin C with or without metformin as above (Figure 2B). Partial to no protection from mitomycin C was observed after metformin pre-treatment across the cell lines (Table 1), indicating that factors other than decreased proliferation were the major contributors to metformin-dependent cisplatin resistance.

**Table 1:** Cytotoxicity in OSCC cells treated with or without metformin and cisplatin or mitomycin C

**Compounds**	**LD**_**50 **_**(μM)**					
	**WHCO1**		**WHCO5**		**SNO**	
Cisplatin	70.88 ± 13.8	p = 0.009	11.68 ± 3.62	p = 0.075	11.01 ± 1.62	p = 0.0001
Met + Cisplatin	126.02 ± 26.57		28.03 ± 15.81		28.16 ± 3.37	
Mitomycin C	32.73 ± 2.49	p = 0.003	32.87 ± 3.03	p = 0.25	9.92 ± 1.80	p = 0.011
Met + Mitomycin C	37.15 ± 0.79		30.19 ± 4.36		16.64 ± 2.77	

OSCC cells were treated with 10 mM metformin (Met) and either cisplatin or mitomycin C and MTT assays performed. LD_50_ (μM) was calculated on replicates (n = 3, mean ± SD).

### Metformin treatment increases lactate production, intracellular NAD(P)H and low molecular weight reduced thiols in OSCC cells

**Figure 4:**
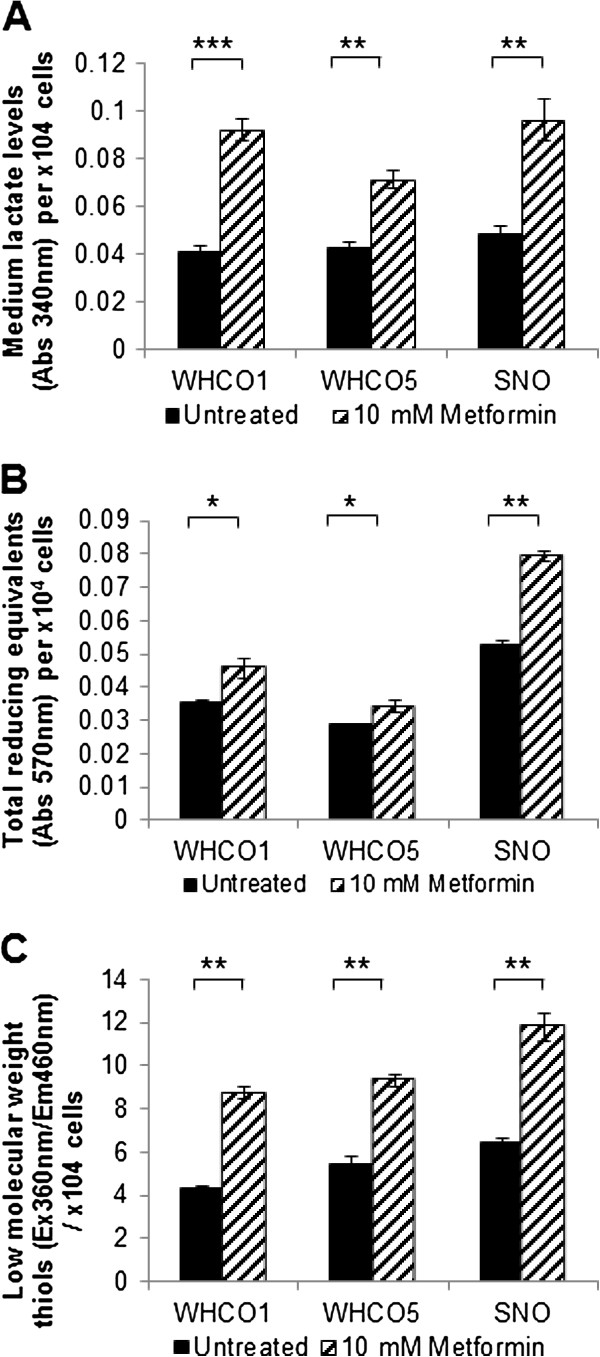
**Metformin increases lactate production, intracellular NAD(P)H and low molecular weight reduced thiols in OSCC cells. A**, Increased secretion of lactate (per 10^4^ cells) indicated increased glycolysis levels in OSCC cells treated with 10 mM metformin for 24 hours in comparison to untreated cells (n = 3, mean ± SD). **B**, Elevated total reducing equivalents (per 10^4^cells) in OSCC cells treated with 10 mM metformin for 24 hours in comparison to untreated cells (n = 3, mean ± SD). **C**, Low molecular weight thiols levels (per 10^4^ cells) is higher in OSCC cells treated with 10 mM metformin for 24 hours, in comparison to untreated cells (n = 3, mean ± SD).

Metformin has been shown to increase cellular glucose transport and glycolytic rates [33]. We hypothesized that such an occurrence in OSCC cells could result in an enhanced intracellular reducing environment (increased NAD(P)H levels) and the potential for increased intracellular reduced thiol levels, thus contributing to the observed metformin-induced protection against cisplatin. Cisplatin cytotoxicity has been previously shown to be antagonized by low-molecular-weight reduced thiols via cisplatin-thiol adduct formation, specifically with glutathione. Glutathione is the major contributor to intracellular thiols, existing in millimolar amounts in the cytosol [7,8,34]. We found that glycolysis (as measured by lactate output), and indirectly, glucose utilization, was indeed significantly increased for all OSCC cell lines after treatment with 10 mM metformin for 24 hours relative to untreated controls (Figure 4A). As predicted, total intracellular NAD(P)H levels (quantified by tetrazolium (MTT) reduction) (Figure 4B) and low-molecular weight thiol levels (monobromobimane-thiol adduct fluorescence) (Figure 4C) were significantly elevated for all OSCC cell lines following metformin treatment relative to untreated controls.

### Intracellular thiols mediate metformin induced cisplatin protection in OSCC cells

**Figure 5:**
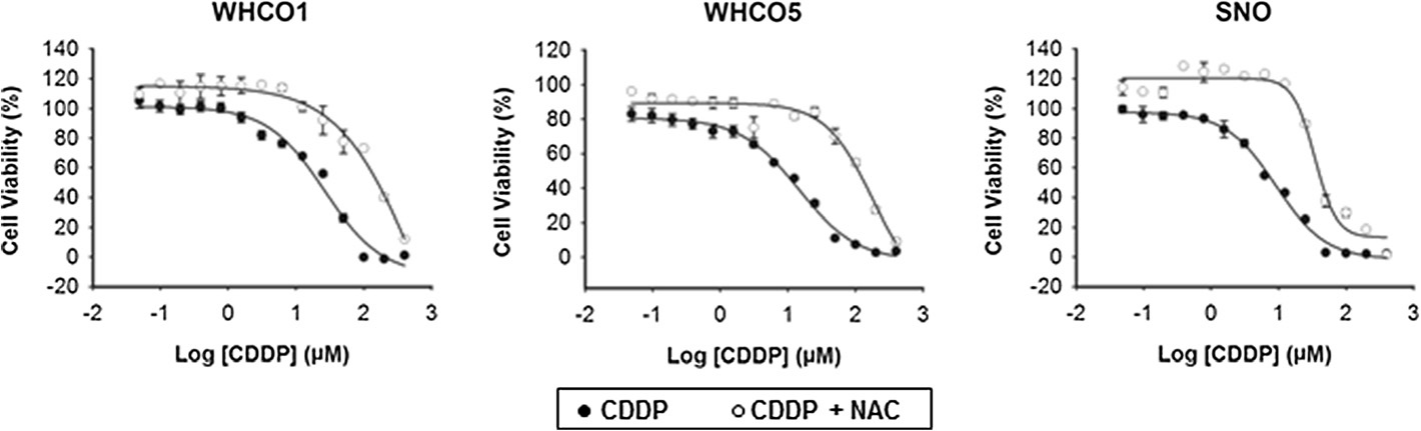
**Increased intracellular thiols causes cisplatin resistance in OSCC cells.** The cell permeable thiol derivative NAC was used to confirm the role of thiols in cisplatin resistance in OSCC cells. Cells were either untreated or treated with 10 mM NAC for 24 hours and then treated with a concentration range of cisplatin for a further 48. Cytotoxicity was assessed by MTT assay. All NAC-cisplatin treated cells displayed higher LD_50_ values than cisplatin treated cells alone (n = 3, mean ± SD).

**Figure 6:**
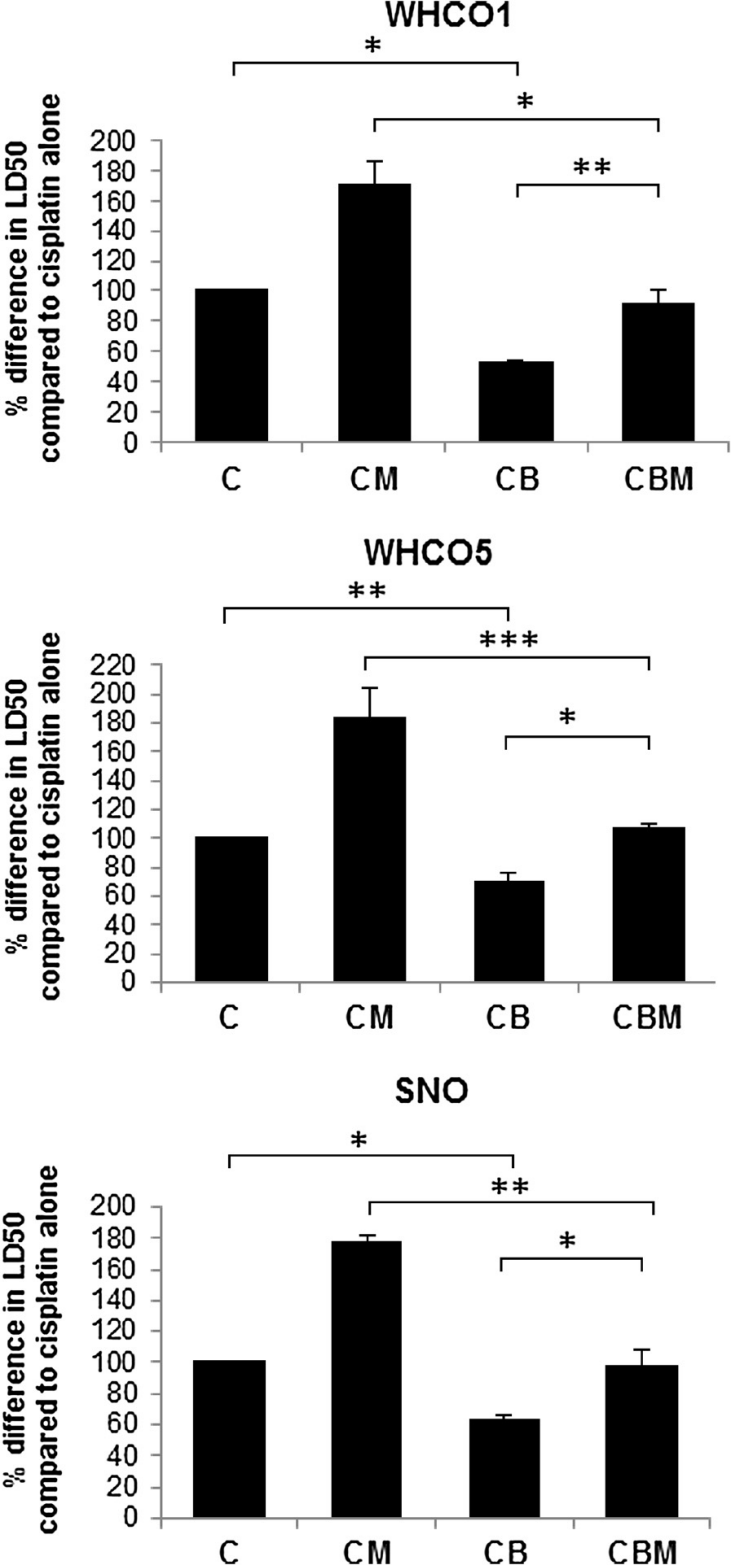
**Metformin-induced cisplatin resistance is reversed by glutathione depletion in OSCC cells.** The glutathione synthesis inhibitor, BSO was used to confirm the involvement of elevated glutathione levels in metformin induced cisplatin resistance in OSCC cells. MTT assays for cytotoxicity were performed as described, with cells treated with cisplatin alone (C), or in the presence 0.4 mM BSO (CB), or metformin and cisplatin (CM), or metformin and cisplatin in the presence of 0.4 mM BSO (CMB). Data is expressed as the percentage difference of LD_50_ values for each treatment relative to cisplatin alone (n = 3, mean ± SD). Predictably, the inhibition of glutathione synthesis increased cisplatin toxicity as LD_50_ values for cisplatin-BSO treated cells were significantly lower than cisplatin alone. Importantly, the presence of the inhibitor ablates the protective effect of metformin, with LD_50_ values for cisplatin-metfomin-BSO treated cells approaching those of cisplatin alone.

To confirm that increased thiol levels can protect OSCC cells against cisplatin, cells were treated with the cell permeable thiol derivative, N-acetyl cysteine (NAC) (10 mM) prior to cisplatin exposure [35]. Predictably, all OSCC cell lines were significantly protected against cisplatin cytotoxicity by NAC pre-treatment (Figure 5). Therefore, our hypothesis, that a metformin-dependent increase in intracellular thiols is primarily responsible for the observed protection against cisplatin, seemed highly plausible. Since glutathione is the major thiol species within the cells, we confirmed its role in metformin-induced cisplatin resistance using the glutathione synthase inhibitor, BSO [32], to deplete intracellular glutathione pools. Cells were treated with metformin in the presence of BSO, prior to cisplatin exposure. Glutathione depletion by BSO almost completely reversed the protective effect of metformin for all OSCC cell lines, confirming the role of reduced-glutathione in metformin-induced cisplatin resistance (Figure 6). We also observed that BSO increased cisplatin cytotoxicity, with lower LD_50_ values, and this was anticipated as decreased intracellular glutathione levels would result in less cisplatin-thiol sequestration and an increase in cisplatin-DNA adduct formation.

### OSCC cells are highly susceptible to copper-bis(thiosemicarbazones)

**Figure 7:**
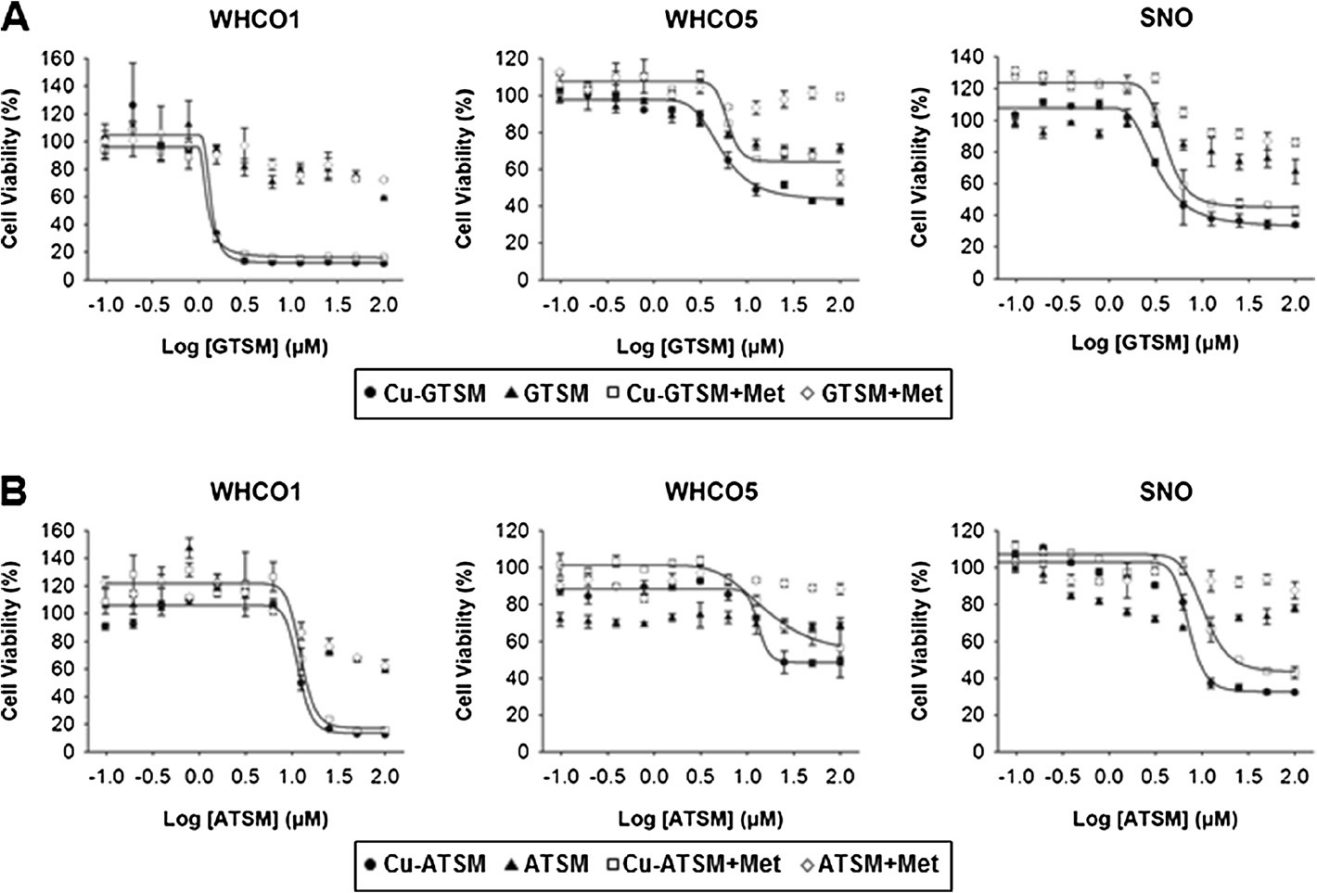
**Copper bis(thiosemicarbazones) are highly toxic to OSCC cells in the presence of metformin.** OSSC cells, untreated or treated with 10 mM metformin for 24 hours and then treated with **(A)** GTSM or Cu-GTSM, or **(B)** ATSM or Cu-ATSM for a further 48 hours were assessed by MTT assay. The non-copper-conjugated bis(thiosemicarbazones) showed relatively little toxicity with LD_50_ values greater than 200 μM in both the presence and absence of metformin. The copper-conjugated compounds however displayed considerable toxicity to OSCC cells with similar LD_50_ values for metformin treated-and untreated compounds for WHCO1, WHCO5, and SNO cells.

Given the above observations, we considered the role of cytotoxic molecules that are compatible with increased intracellular reducing conditions and could therefore be used in conjunction with metformin. In this way, the cytostatic effects of metformin could be utilised when combined as an adjuvant in chemotherapy regimens; since there is also evidence that metformin targets cancer stem cells, this would offer a considerable added advantage [18]. The copper bis(thiosemicarbazone) derivatives ATSM and GTSM have been previously shown to be trapped intracellularly under reducing conditions [22]. We therefore tested their efficacy as cytotoxic agents against OSCC cell lines with or without metformin. OSCC cells were pre-treated with or without 10 mM metformin for 24 hours and then treated with copper-bis(thiosemicarbazones) and 10 mM metformin for 48 hours (Figure 7). Interestingly, we found that both Cu-ATSM and Cu-GTSM displayed significant cytotoxicity for all cell lines, both in the presence and absence of metformin treatment, with LD_50_ values lower than or comparable to cisplatin alone. Cu-GTSM displayed lower LD_50_ levels than Cu-ATSM (Table 2). Statistically there was no difference between untreated and metformin treated samples (p > 0.05). Non-copper-conjugated bis(thiosemicarbazone) compounds displayed far lower levels of cytotoxicity than their copper-conjugated counterparts, with LD_50_ concentrations over 200 μM; copper alone had minimal effect on cells at the concentrations used in this study.

**Table 2:** Cytotoxicity in OSCC cells treated with or without metformin and with Cu-GTSM or Cu-ATSM

**Compounds**	**LD**_**50 **_**(μM)**		
	**WHCO1**	**WHCO5**	**SNO**
Cu-GTSM	1.14 ± 0.16	5.39 ± 0.9	3.37 ± 0.23
Metformin + Cu-GTSM	1.16 ± 0.12	6.24 ± 0.01	4.23 ± 0.7
GTSM	>200	>200	>200
Metformin + GTSM	>200	>200	>200
Cu-ATSM	11.93 ± 0.47	13.51 ± 0.81	7.099 ± 0.57
Metformin + Cu-ATSM	12.54 ± 0.19	12.05 ± 0.83	10.90 ± 0.77
ATSM	>200	>200	>200
Metformin + ATSM	>200	>200	>200

OSCC cells were treated with 10 mM metformin and either GTSM, Cu-GTSM, ATSM or Cu-ATSM and the MTT assay performed. LD_50_ (μM) was calculated on replicates (n = 3, mean ± SD).

## Discussion

We have established that metformin significantly reduces the proliferation of OSCC cells. However, we observed that metabolic alterations caused by metformin rendered cells less sensitive to the commonly used chemotherapeutic agent, cisplatin. Previous studies have shown that metformin can reduce sensitivity to cisplatin through the activation of pro-survival signals via Akt [20] and hyperactivation of Akt has been linked to increased glycolysis [36]. Those studies therefore support our findings, which show that metformin increases glycolysis with a subsequent increase in intracellular reducing equivalents and a concomitant increase in intracellular reduced thiols.

Since cisplatin is ineffective in a reducing intracellular environment, our findings also support observations regarding cisplatin chemoresistance in tumours; cancer cells within the tumour are known to display a highly reducing phenotype and resist cisplatin chemotherapy [37]. However, in recent years, the observation that tumours consist of cells in differing metabolic states to surrounding normal tissue [38] has encouraged the concept of cancer-cell specific metabolic targeting as an increasingly popular strategy in cancer therapy [39]. Our study highlights the use of metformin with cytotoxic agents that are compatible with or remain active under reducing conditions, thus paving the way for novel drug therapy combinations for the treatment of this highly aggressive malignancy.

Mitomycin C, which must be reductively activated to exert its biological effects [40], is one potential candidate for this strategy as partial to no protection from this drug was observed after metformin pre-treatment. However, an obvious concern with the use of mitomycin C and related DNA crosslinkers in combination with metformin would be the potential for decreased drug effectiveness or the emergence of drug resistance *in vivo,* due to the anti-proliferative effects of the biguanide. Therefore, agents that are either reductively activated or tolerant, and that target proliferating and non-proliferating tumour cells, would be a more logical choice for use in combination with metformin in OSCC. We have established that a potential highly efficacious combination strategy of this kind, could be metformin and the copper-bis(thiosemicarbazones), Cu-GTSM or Cu-ATSM. Bis(thiosemicarbazones) have been considered for cancer treatment since the 1950’s [23], whilst the copper-bis(thiosemicarbazones) have been shown to possess potent anti-cancer activities and are attractive candidates for use as chemotherapeutics as they often preferentially accumulate in tumour tissue and are retained in cells under reducing conditions [22]. We have shown that Cu-ATSM and Cu-GTSM, in contrast to non-copper conjugated bis(thiosemicarbazones), are highly cytotoxic to OSCC cells, both in the presence and absence of metformin, and are thus metformin-compatible. The fact that an increase in toxicity was not observed for Cu-ATSM or Cu-GTSM in the presence of metformin suggests that: (1) there already exists a sufficiently high intracellular reducing environment in the OSCC cell lines used (a common observation in cancer cells [37]) to allow for the intracellular accumulation of these compounds to toxic levels, and (2) that the mechanisms of toxicity of these compounds, are compatible with, but not necessarily dependent on a intracellular reducing environment.

Predictably we found that Cu-GTSM exhibited lower LD_50_ values than Cu-ATSM as Cu-GTSM is known to be rapidly reduced by intracellular thiols resulting in cell retention, copper release and ultimately apoptosis via oxidative stress, and/or the inhibition of DNA synthesis and oxidative phosphorylation [41,42]; Cu-ATSM on the other hand has been shown to be poorly reduced by intracellular thiols and thought to be maintained in a reduced state (and thus retained intracellularly) only under hypoxic conditions [41]. Recently, however, Donnelly *et al.* have shown that Cu-ATSM can be retained in cells under normoxic conditions when the intracellular reducing environment is increased due to factors such as impaired mitochondrial electron transport chain function [22]. These findings appear to agree with the findings of our study as SNO cells, which exhibit the greatest intracellular reducing environment (in the absence of metformin) of all the OSCC cell lines tested (Figure 4B), exhibit the greatest sensitivity to Cu-ATSM (Table 2). Nonetheless, the fact that all OSCC cell lines were highly sensitive to Cu-ATSM alone or in combination with metformin, at LD_50_ values comparable to or lower than those for cisplatin for all OSCC cell lines used, is extremely promising given its increased stability over Cu-GTSM and investigatory Food and Drug Administration approval of ^64^Cu-ATSM for use as a hypoxia contrast agent [43].

## Conclusions

Metformin, which has an extensive track record and is well tolerated by individuals, has been shown to suppress cancer cell proliferation. We have established that metformin significantly reduces cell proliferation in OSCC cell lines. However, we found metformin causes resistance to cisplatin in OSCC cell lines and as such we advise that caution be used when administering cisplatin to diabetic patients treated with metformin and in the use of metformin as an adjuvant to cisplatin chemotherapy. Furthermore, we have shown that two copper-conjugated bis(thiosemicarbazones), Cu-ATSM and Cu-GTSM, exhibit marked cytotoxicity in OSCC cells in the presence of metformin. The preliminary data presented in this study justifies further investigations into the therapeutic effects of copper-bis(thiosemicarbazones) in both the presence and absence of metformin, for OSCC. In addition metformin lends itself to combination therapy with reduction compatible or activated compounds (unlike cisplatin) for both OSCC and potentially other cancers where similar metabolic changes are observed.

## Abbreviations

BSO: Buthionine sulfoximine; Cu-ATSM: Copper diacetyl-bis(4-methylthiosemicarbazonato)copper(II); Cu-GTSM: Copper glyoxal-bis(4-methylthiosemicarbazonato)copper(II); DMSO: Dimethyl Sulfoxide; FCS: Foetal calf serum; ICP-MS: Inductively coupled plasma mass spectrometry; LDH: Lactate dehydrogenase; NAC: N-acetyl-L-cysteine; MTT: 3-(4,5-dimethylthiazol-2-yl)-2,5 diphenyltetrazolium bromide.

## Competing interests

Authors LHD and DMD are co-applicants on a patent for the treatment of cancer and OSCC using metformin and copper-conjugated compounds. The authors declare that they have no competing interests.

## Authors’ contributions

LHD conceived the study, participated in its design, performed low-molecular weight thiol quantification, synthesized bis(thiosemicarbazone) compounds, conducted data analysis and interpretation, and assisted in drafting the manuscript; RJ carried out the cell proliferation studies, cell cycle analysis, prepared samples for ICP-MS analysis, and contributed to the MTT and LDH assays and statistical analysis; AR assessed bis(thiosemicarbazone) structures by NMR spectroscopy; RV participated in manuscript preparation; DMD conceived the study, participated in its design, acquired data for the MTT, LDH, thiol, reducing equivalents, and glutathione depletion assays, conducted data analysis and interpretation, and drafted the manuscript. All authors read and approved the final manuscript.

## Pre-publication history

The pre-publication history for this paper can be accessed here:

http://www.biomedcentral.com/1471-2407/14/314/prepub
